# Specialty choice in UK junior doctors: Is psychiatry the least popular specialty for UK and international medical graduates?

**DOI:** 10.1186/1472-6920-9-77

**Published:** 2009-12-24

**Authors:** Seena Fazel, Klaus P Ebmeier

**Affiliations:** 1 University of Oxford, Department of Psychiatry, Warneford Hospital, Oxford OX3 7JX, UK

## Abstract

**Background:**

In the UK and many other countries, many specialties have had longstanding problems with recruitment and have increasingly relied on international medical graduates to fill junior and senior posts. We aimed to determine what specialties were the most popular and desirable among candidates for training posts, and whether this differed by country of undergraduate training.

**Methods:**

We conducted a database analysis of applications to Modernising Medical Careers for all training posts in England in 2008. Total number of applications (as an index of popularity) and applications per vacancy (as an index of desirability) were analysed for ten different specialties. We tested whether mean consultant incomes correlated with specialty choice.

**Results:**

In, 2008, there were 80,949 applications for specialty training in England, of which 31,434 were UK graduates (39%). Among UK medical graduates, psychiatry was the sixth most popular specialty (999 applicants) out of 10 specialty groups, while it was fourth for international graduates (5,953 applicants). Among UK graduates, surgery (9.4 applicants per vacancy) and radiology (8.0) had the highest number of applicants per vacancy and paediatrics (1.2) and psychiatry (1.1) the lowest. Among international medical graduates, psychiatry had the fourth highest number of applicants per place (6.3). Specialty popularity for UK graduates was correlated with predicted income (p = 0.006).

**Conclusion:**

Based on the number of applicants per place, there was some consistency in the most popular specialties for both UK and international medical graduates, but there were differences in the popularity of psychiatry. With anticipated decreases in the number of new international medical graduates training in the UK, university departments and professional associations may need to review strategies to attract more UK medical graduates into certain specialties, particularly psychiatry and paediatrics.

## Background

A number of countries have increased medical school places to address shortages in the amount of graduates training in certain specialties [1-3]. To make up the shortfall, doctors who have trained overseas have increasingly worked in health care systems in many Western countries. For example, in the UK, 24% of consultants (the equivalent of attending physicians) appointed in 1992-2001 had trained abroad, a proportion that has increased since the 1960s [4].

In the UK, the introduction of a new national application system for medical specialties in 2007, Modernising Medical Careers (MMC), was designed in part to redress the uneven demand for medical specialties by introducing a national competition for places. Using MMC data for applications for training posts in 2008, we examined the specialty choices of UK compared with international medical graduates (IMGs). We aimed to determine which specialties were the most popular and desirable, and whether this differed by country of training. Our hypotheses were related to the known preferences of medical students for specialty training [5], but as the national market in training places is now subject to international competition, we predicted that the choice of specialty would also depend on country of origin, thus leading to predictable trends in the relative contribution of overseas graduates to different specialties in the National Health System. In particular, we hypothesised that specialty choice would be associated with predicted income, as it has been demonstrated for all specialties in the US [6], particularly if candidates perceived no need for strategic applications for less attractive disciplines.

## Methods

### Modernising Medical Careers (MMC): Analysis of the national database

MMC http://www.mmc.nhs.uk/ is the national clearing house for all post-graduate medical specialty training applications. It was set up in 2007 and is administered as an office of the UK Department of Health in London. In 2008, applications were locally led, and individuals could apply to as many specialty choices as they wishes, apart from obstetrics and gynaecology and general practice where the number of applications was limited nationally. Information about the country of medical school is collected as part of the application form. We defined IMGs as those whose medical school education was completed outside the UK. We requested and received summary information about all applications for all training grades and specialties in England in 2008, including the number of advertised posts in English Deaneries and the fill rate for each specialty by training grade.

### Sensitivity analysis

To compare competition levels for the estimated economical attractiveness of medical specialty, we also requested average consultant incomes broken down by specialty based on payments made to staff in the three months July to September 2008 from the Workforce Analysis Team at the NHS Information Centre http://www.ic.nhs.uk/. As UK consultant salaries are determined centrally and not thought to vary markedly, means were considered to be the best estimate of average incomes. A plausible hypothesis was that the attractiveness of specialty training, estimated as number of applications per available post, would be influenced by the predicted financial rewards. We tested therefore whether average consultant incomes were correlated with specialty choice in UK graduates compared with IMGs.

### Statistical Analysis

We performed chi-square tests to examine differences in the choices of specialty by country of medical training (UK vs. IMG). We grouped specialties according to the following: Accident and Emergency (Acute Care, Anaesthesia, Emergency Medicine); Medicine (Acute Medicine, Allergy, Audiological Medicine, Cardiology, Clinical Oncology, Clinical Pharmacology, Core Medical Training, Dermatology, Endocrinology & Diabetes, Gastroenterology, General Internal Medicine, Genito-Urinary Medicine, Geriatric Medicine, Haematology, Immunology, Infectious Diseases, Medical Oncology, Neurology, Neurophysiology, Occupational Medicine, Oncology, Palliative Care, Palliative Medicine, Rehabilitation Medicine, Renal Medicine, Respiratory Medicine, Rheumatology, Tropical Medicine); Laboratory Medicine (Chemical Pathology, Clinical Genetics, Clinical Neurophysiology, Histopathology, Medical Microbiology, Virology); General Practice; Psychiatry; Paediatrics (which included Paediatric Cardiology); Public Health; and Surgery (which included Trauma and Orthopaedics). We used a large grouping for Medicine as applications at more junior levels are for core medical training, and subspecialties are chosen after some years of this core training. We also examined trends for specialty by training seniority in Psychiatry. Spearman's rank correlation (r_s_) was used to examine if average income of specialty was correlated with choice. Statistical analysis was done with StatsDirect (Version 2.7.2; Altrincham, Cheshire, WA14 4QA, UK) and SPSS (Version 17.0.1; SPSS Inc. Chicago, Illinois 60606, USA).

## Results

There were 10,243 training posts advertised through MMC in 2008, of which 9,172 (90%) were filled. There were 80,949 applications by doctors for these posts, of whom 49,515 (61%) were IMGs.

### Workforce planning

In 2008, medicine had the highest number of training places (n = 2686, 26%), followed by General Practice (n = 2384, 23%), and Surgery (n = 1289, 13%). Psychiatry had 944 posts, which represented 9% of all training posts (see Table 1 for all specialties). The fill rate of posts varied significantly from 78% in Accident and Emergency to 101% in General Practice (Table 1). Psychiatry filled 91% of posts advertised.

**Table 1:** Filled and vacant places for advertised jobs in specialty groups in order of total number of places advertised*

Specialty Group	Filled	Remained Vacant	Total places advertised (% filled)^2^	% of Total
**Medicine**	2,308	378	2,686 (86%)	26%

**General Practice**	2,417	0	2,384 (101%)	23%

**A & E**	1,099	303	1,402 (78%)	14%

**Surgery**	1,122	167	1,289 (87%)	13%

**Psychiatry**	862	82	944 (91%)	9%

**Paediatrics**	710	74	784 (91%)	8%

**Obst & Gynae**	290	73	363 (80%)	4%

**Radiology**	175	1	176 (99%)	2%

**Lab. Medicine**	113	24	137 (82%)	1%

**Public Health**	76	2	78 (97%)	1%

**Total**	**9,172**	**1,071**	**10,243 (90%)**	**100%**

* MMC 2008 Specialty Training Recruitment Monitoring as recorded on 29^th ^October 2008

^2 ^Percentage of advertised places that were filled (if >100%, more candidates were appointed than places advertised)

A&E = Accident and Emergency, incl. Anaesthetics; Lab. Medicine = Laboratory Medicine incl. Neurophysiology; Obst & Gynae = Obstetrics & Gynaecology

### What were the most attractive specialties for UK graduates and International Medical Graduates (IMGs)?

**Figure 1:**
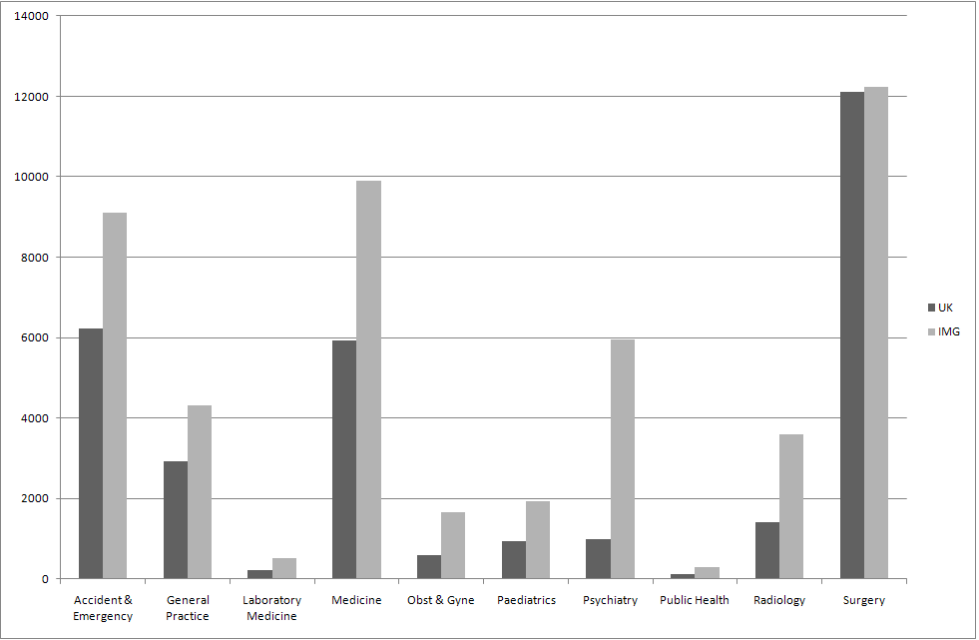
**Applicants to medical specialty training places in England, separated by country of graduation (UK versus International Medical Graduate [IMG])**.

The number and proportion of applicants with UK and international medical qualifications varied significantly between the different specialty groups: in Surgery, the proportion of UK graduates was highest at 50%; in Psychiatry, it was lowest at 14% (Figure 1; Table 2). There was no difference in proportion of IMG applications depending on seniority in Psychiatry (χ^2^_3 _= 1.8; p = 0.35).

**Table 2:** UK and International Medical Graduate (IMG) applicants (country of qualification) for vacancies in specialty groups in order of total number of applicants*

Specialty Group	UK (%)^1^	IMG (%)^1^	Total (% UK applicants)^2^
**Surgery**	12,105 (39%)	12,225 (25%)	24,330 (50%)

**Medicine**	5,917 (19%)	9,898 (20%)	15,815 (37%)

**A & E**	6,226 (20%)	9,116 (18%)	15,342 (41%)

**General Practice**	2,924 (9%)	4312 (9%)	7,236 (40%)

**Psychiatry**	999 (3%)	5,953 (12%)	6,952 (14%)

**Radiology**	1,415 (5%)	3,588 (7%)	5,003 (28%)

**Paediatrics**	936 (3%)	1,941 (4%)	2,877 (33%)

**Obst & Gynae**	587 (2%)	1,668 (3%)	2,255 (26%)

**Lab. Medicine**	212 (1%)	514 (1%)	726 (29%)

**Public Health**	113 (0.4%)	300 (1%)	413 (27%)

**Total**	**31,434**	**49,515**	**80,949 (39%)**

* MMC 2008 analysis of the 2008 Medical Recruitment Specialty Preferences

^1^ Percentage of applicants who applied for specialty

^2 ^Percentage of applicants who graduated in the UK

A&E = Accident and Emergency, incl. Anaesthetics; Lab. Medicine = Laboratory Medicine incl. Neurophysiology; Obst & Gynae = Obstetrics & Gynaecology

As an index of relative popularity, we examined the absolute number of applications for different specialties (Figure 1). Psychiatry was sixth most popular specialty for UK graduates, ahead of Paediatrics, Obstetrics and Gynaecology, Laboratory Medicine and Public Health. For IMGs, Psychiatry was the fourth most popular. We found that the numbers of applicants from UK graduates and IMGs were highly correlated across subject groups (r_s _= 0.95).

As an index of relative desirability, we computed applications per vacancy in each specialty for UK and IMGs separately (Table 3). For UK graduates, Surgery (9.4 applications per vacancy) and Radiology (8.0) were the most desirable, while Paediatrics (1.2) and Psychiatry (1.1) had the least number of applications per vacancy. Among IMGs, the pattern was different: Radiology (20.4 applications per vacancy) and Surgery (9.5) remained the most desirable, while Public Health (3.9), Laboratory Medicine (3.8), and Paediatrics (2.5) had lowest applicant rates. Psychiatry was the fourth most desirable specialty (6.3 applicants per vacancy) among IMGs.

**Table 3:** UK and International Medical Graduate (IMG) applicants per vacancy and current total consultant earnings in order of UK applicants per vacancy*

Specialty Group	UK Applicants per Vacancy^1^	IMG Applicants per Vacancy^2^	Mean Total Earnings per Full Time Equivalent^3^
**Surgery**	9.39	9.48	£123,100

**Radiology**	8.04	20.39	£119,600

**A & E**	4.44	6.50	£117,750

**Medicine**	2.20	3.69	£118,400

**Obst & Gynae**	1.62	4.60	£119,300

**Lab. Medicine**	1.55	3.75	£118,400

**Public Health**	1.45	3.85	£109,200

**Paediatrics**	1.19	2.48	£115,700

**Psychiatry**	1.06	6.31	£108,400

**All**	**3.07**	**4.83**	**£116,650**

*Source: Information Centre for Health and Social Care 2008 NHS Staff Earnings Estimates, July to September 2008

^1 ^Spearman's rank correlation (r_s_) with Mean Total Earnings: r_s _= 0.85, p = 0.006; ^2^r_s _= 0.44, p = 0.25;

^3 ^Calculated as mean basic salary, but for all earnings. This includes basic salary, plus hours related pay, overtime, occupation payments, and location payments.

A&E = Accident and Emergency, incl. Anaesthetics; Lab. Medicine = Laboratory Medicine incl. Neurophysiology; Obst & Gynae = Obstetrics & Gynaecology

### Associations with predicted incomes

We examined the correlation between mean income levels by consultants in each specialty and their applicants per place by UK graduates and IMGs. Estimated annual NHS consultant salaries within the specialty groups varied from £108 k (Psychiatry) to £123 k (Surgery). Expected consultant salary correlated highly with specialty popularity in UK graduates, but not in IMGs (r_s _= 0.85, p = 0.006 in UK graduates; r_s _= 0.44, p = 0.25 in IMGs).

## Discussion

This analysis of all English applications for specialty training in 2008 drew on data from a new system in the UK, Modernising Medical Careers (MMC). As part of MMC, information was collected on medical school of origin of all applicants, and this enabled us to assess any differences in specialty choice between UK graduates and IMGs. Previous work on specialty preference has been based on surveys of UK medical school graduates, and thus not been able to assess the impact of overseas applicants [5]. In addition, this is the first study, to our knowledge, that has looked at the popularity of specialty training in the UK using nationally collected data on all applications.

The findings on Psychiatry were notable. The first was Psychiatry had the highest proportion of overseas applicants at 86%. Second, the number of applications per training place, a marker of specialty desirability, was lowest for Psychiatry among UK graduates. Psychiatry has had longstanding problems with recruitment in the UK [7,8] and other countries [9-13]. As a consequence, IMGs have increasingly met the shortfall in recruitment. In the UK, for example, around 25% of consultant psychiatric appointments during 1964-2001, a proportion higher than most other specialties, qualified abroad [4], and in 2008, the proportion of UK graduates who took the membership examination of the Royal College of Psychiatrists was 6% [14].

A number of implications arise from the large proportion of overseas applicants to Psychiatry. The first relates to the potential impact of the loss of qualified doctors on the medical systems of the country of origin of these international graduates [15]. Second, ethnic stereotyping, particularly negative perceptions of individuals from ethnic minorities, which has been found at some UK medical schools, will increasingly need to be addressed in postgraduate training [16]. A final implication is the observation that IMGs are more likely to fail this membership examination than UK graduates, so the number of well-trained specialists for future consultant posts will need to be considered in national workforce planning. Furthermore, other work has shown that IMGs face challenges understanding health care systems and local pharmaceuticals that may impact of their performance [17] (which the authors attribute partly to the fact that many IMGs will have chosen psychiatry as a second career option). This situation in the UK is in contrast to other western countries, such as the US, where the proportion of these overseas graduates taking up psychiatric residency has been estimated to be no more than 30% [1]. However, the US data is based on those successfully taking up training posts in contrast to our data that is derived from applications. Psychiatry was the least desirable specialty for UK applicants. A number of studies have explored the reasons for this including the perceptions that it lacks evidence-based treatments, the prognosis of patients is poor, and the lack of respect from other medical colleagues [7,8,18]. There were 999 applications to Psychiatry from UK medical graduates, representing 3.2% of all applications from UK graduates, a proportion that appears to have decreased compared with a national survey of medical graduates in 2002, where it was 3.8% [19].

Another specialty with low numbers of applicants per place was Paediatrics which had the second lowest proportion of applicants per place among UK applicants and the lowest proportion among IMGs. In the US, barriers to careers in Paediatrics have included perceptions that it is associated with lower incomes, longer hours, more on call, and high rates of burnout [20]. In the UK, in a large study of nine cohorts of UK medical graduates, six factors were rated as influential by significantly lower percentages of those who chose paediatrics than those who selected other specialties: the doctors' current domestic circumstances, anticipated hours and working conditions in the specialty, future financial prospects, career and promotion prospects, experience of jobs so far and advice from others [21].

Our finding that specialty popularity was not correlated with the mean consultant income in that specialty for IMGs may be the consequence of a number of factors. Factors that may be relevant in driving these doctors from their countries include hierarchical and inflexible medical careers. A specific interest in certain specialities, such as Psychiatry, or a lack of a materialistic attitude seem less likely an explanations for the different behaviour of IMGs. Other factors including the influence of undergraduate medical education on specialty choice may be relevant and, in some individuals, a reduced confidence to compete in the more desirable specialty groups may be important when the priority is to find any training post at all. Nevertheless, one would assume that motivations for subject choice are similar in principle between UK trainees and IMGs. If IMGs opt for Psychiatry, that may just reflect the availability of vacancies (and relative chances of success), given that the attractions of training in the UK are still the same for Psychiatry. The vastly different salaries in the UK compared with some overseas countries may also mean that IMGs do not find differential amounts of pay important in determining their choice.

This study is limited by the use of data from one country and from one year, and is based on applications. The impact of the new medical schools and the graduate entry programmes will not have fully materialised by 2008, and it is possible that application patterns may change in subsequent years. However, evidence from a large survey of graduate entrants showed little difference in specialty choice between graduate and non-graduate entrants to UK medical schools, with a modest increase in the number who wish to train in General Practice [22]. In addition, other factors that we were not able to assess may have stronger associations with specialty choice than income, such as lifestyle [6] and personality [23]. We did not examine the data by gender, and future research could investigate if there are important differences between male and female medical graduates in determining specialty choice. Research in the US has found patterns of specialty popularity similar to the ones we report, with Psychiatry the least popular medical specialty for final year medical students after Pathology [6], and in Jordan, Psychiatry was among the least popular specialties [24].

The 57% increase in UK medical graduates from 1998 to 2005 has been welcomed as a positive move to address workforce planning issues [3], which partly may have been a response to the fact that the National Health Service was relying on IMGs to meet the shortfall in junior doctor recruitment. Although some of the new medical schools have focused on community based learning and more behavioural sciences in their curricula [3], an important contribution they can make is to redress the unattractiveness of some medical specialties and meet the new challenges of chronic diseases for medicine in the coming decades [25]. In addition, our data suggest that for some specialties, such as Psychiatry [26] and Paediatrics, professional associations, university departments, and others involved in medical education need to consider how best to increase recruitment and retention of trainee doctors.

## Conclusion

This analysis of all 80,949 applications for medical training in England found large differences between specialties in terms of popularity and desirability, which were mostly consistent depending on whether the applicant was a UK graduate or an IMG. Based on applicants per place, Psychiatry and Paediatrics were the least popular broad specialty groupings for UK medical graduates. Improving recruitment and retention of trainee doctors in certain specialties remain significant issues for consideration by medical schools and professional associations.

## Competing interests

The authors declare that they have no competing interests.

## Authors' contributions

SF and KPE devised the study, interpreted the findings, drafted the article, critically revised it, and approved the final version. KPE conducted the analysis.

## Pre-publication history

The pre-publication history for this paper can be accessed here:

http://www.biomedcentral.com/1472-6920/9/77/prepub
